# The Effect of Abdominal Massage with Extra-Virgin Olive Oil on Constipation among Elderly Individuals: A Randomized Controlled Clinical Trial

**DOI:** 10.30476/ijcbnm.2021.88206.1495

**Published:** 2021-10

**Authors:** Amir Faghihi, Sied Saeed Najafi, Mohammad Hashem Hashempur, Majid Najafi Kalyani

**Affiliations:** 1 Student Research Committee, School of Nursing and Midwifery, Shiraz University of Medical Sciences, Shiraz, Iran; 2 Department of Medical Surgical Nursing, School of Nursing and Midwifery, Shiraz University of Medical Sciences, Shiraz, Iran; 3 Noncommunicable Diseases Research Center, Fasa University of Medical Sciences, Fasa, Iran

**Keywords:** Complementary and alternative medicine, Constipation, Massage, Olive oil, The elderly

## Abstract

**Background:**

Constipation is one of the most prevalent problems during old age. Abdominal massage is a complementary method in controlling constipation. This study is conducted with the aim of determining the effect of abdominal massage with extra-virgin olive oil on constipation among the elderly.

**Methods:**

In this single-blind randomized controlled clinical trial, 54 old individuals in Shiraz nursing homes during November 2018-March 2019 were selected randomly and then allocated to three groups of 18, based on block randomization. The first group underwent abdominal massage with olive oil for five consecutive days (each day one time for 15 minutes). The second group underwent abdominal massage with water similar to the first group. No specific intervention was applied to the control group. All three groups received their medical treatment. The constipation scores were examined using constipation assessment scale (CAS) before the intervention and on the sixth day. Data analysis was done through SPSS 22 using Chi-square, paired t-test, and ANOVA. P<0.05 was considered as the level of statistical significance.

**Results:**

There were no statistically significant differences among the three groups before the intervention. The results demonstrated that the mean score of constipation further decreased in the olive oil group (5.62±1.89 to 2.06±0.99) (P≤0.001) than the massage with water (5.05±1.25 to 3.11±0.99) (P=0.02), and the control group (4.44±1.38 to 5.22±1.35) (P=0.006).

**Conclusion:**

Due to the greater effectiveness of abdominal massage with extra-virgin olive oil, the use of this method is recommended in treatment of constipation among the elderly.

**Trial Registration Number:**

IRCT20180923041101N

## INTRODUCTION

With the increase in life expectancy as well as improvements made in treatment methods, the world’s population is becoming old. ^1^ The world’s population increases by 1.7% every year, while the same rate is 2.5% for ages of 65 years and above. Thus, it has been predicted that the world’s above-65-year-old population will double by 40 years from now. Among this population, 52% will belong to Asian countries and 40% to developed ones. ^2^ Previous studies have demonstrated that 80% of the elderly people suffer from at least one chronic disease, which made them prone to the risk of disability and death. ^3^

Constipation is one of the most prevalent digestive problems. ^4^ The prevalence of constipation is different in various countries due to variations in lifestyle and diet. ^5^ Research has indicated that the prevalence of this disorder was 8-26% in Europe, with a mean of 22.3%. ^6^ In the studies conducted in Iran, the prevalence of constipation has been reported to vary from 3.5% to 32.9%. ^7^ As an individual becomes older, the prevalence of constipation increases incredibly. In this context, the prevalence of constipation has been reported to be 34% among females and 26% among males above 84 years of age. With increase in age, this value increased as well. ^8^

Constipation can be a symptom of a disease or a disease, and requires examination and consideration due to its long-lasting complications and effects on the patients’ life quality. ^9 - 11^ Because of the physiological phenomena relevant to old age, immobility, abundant use of medications, decrease in blood pressure, and patients’ ignorance, constipation may be accompanied with more complications and troubles in the elderly individuals. ^12 , 13^ Constipation influences different aspects of the elderly people’s lives, including the quality of life. ^14^ Different pharmaceutical and non-pharmaceutical methods have been used for treatment of constipation. ^15^ Although pharmaceutical methods make up the commonest method of treatment, long-term use of medications is accompanied by several complications. ^16 , 17^ Given the problems and complications resulting from pharmaceutical treatment, the tendency to use non-pharmaceutical methods, such as complementary medicine and traditional medicine, has increased. ^18 - 20^ Abdominal massage is a non-pharmaceutical method for controlling and treating constipation. ^5 , 21 , 22^ Several studies have addressed the effect of abdominal massage as an inexpensive and non-aggressive method for treatment of constipation. ^23 - 25^ In many studies, traditional oils for abdominal massage have been used for patients with constipation. ^26 , 27^ In Traditional Persian Medicine, application of oil onto the abdomen around the navel was found to be appropriate for treatment of constipation. ^28^ One of the oils used in Traditional Persian Medicine is olive oil, which has been demonstrated to be effective in treatment of skin diseases including psoriasis, and reduction of heart diseases including atrial fibrillation. ^29 - 31^ Extra-virgin olive oil is a type of unrefined olive oil, which is different from other types of olive oil since it is golden green, and tastes mildly hot. ^32^

Given the scarcity of studies conducted on the effect of abdominal massage with olive oil and considering evidence with regard to the use of abdominal massage with olive oil for controlling and treating constipation in Traditional Persian Medicine manuscripts, this study aims to investigate the effect of abdominal massage using extra-virgin olive oil on constipation among elderly individuals.

## MATERIALS AND METHODS

This single-blind randomized controlled clinical trial was conducted during November 2018- March 2019 on the residents of nursing homes in Shiraz, Iran who were selected by simple random sampling. The sample size in this study was calculated based on the previous study. ^12^ In this calculation, according to the constipation score in the studied groups and considering the error of 1% (α=0.01), power of 90% (β=0.10), μ_1_=34.37, μ_2_=11.0, σ_1_=25.77, σ_2_=11.58 and use from the formula for comparing the two means, 54 people (18 people per group) was estimated by MedCalc software by using the following formula:

$n=\frac{\left(z_{1-\alpha /2}+z_{1-\beta })(\sigma_{1}^{2}+\sigma_{2}^{2}\right)}{{\left(\mu_{1}-\mu_{2}\right)}^{2}}$

$n=\frac{{\left(2.57+1.281\right)}^{2}(25.77^{2}+11.58^{2})}{{\left(\mathrm{34.37}-\mathrm{11.0}\right)}^{2}}$

The inclusion criteria of the study were being aged above 60 years (chronological elderly people), obtaining scores >1 based on the constipation assessment scale (CAS), emptying the bowel less than three times a week, and being willing to participate in the study. The exclusion criteria of the study were suffering from cognitive problems (dementia), having a history of surgery in the abdominal area, having an inflammation or open wound around the massage area, suffering from a skin disorder or scar in the abdomen, having a special underlying disease, and consuming other medications which have an interaction with the study goals. 

After the methodology was explained and written consent for participation in the study was obtained, the individuals were randomly allocated to three groups of massage with extra-virgin olive oil, massage with water, and control (each containing 18 patients). This was carried out based on permuted block randomization (ABC) using a random number generator. By default, A: massage with extra-virgin olive oil, B: massage with water, and C: control group. Then, among the random numbers from one to 54, based on the random table, the numbers were taken from the table and randomly divided into three groups. 

In the abdominal massage with extra-virgin olive oil group, the intervention was carried out by a trained and skilled masseur according to the abdominal massage guidelines introduced by the National Health System. ^33^ Abdominal massage with olive oil was performed using 20 milliliters of extra-virgin olive oil for 15 minutes at a specific time period (8-11 AM) for five consecutive days in different nursing homes. ^26^ In this method, abdominal massage was carried out at a 2-4 cm depth by a common masseur for males and by a common masseuse for females. This was done through eight steps as follows:

1- Three movements from the pubic symphysis to the navel

2- Three movements from the two sides of the pelvis toward the pubic symphysis

3- Right wise rotation from the pubic symphysis to the surroundings

4- Small rotation in the left lower abdomen

5- Repetition of step 4 in higher parts and the entire abdomen

6- Repetition of steps 4 and 5

7- Horizontal movement of the abdomen from left to right

8- Vibrations at all above points. ^33^

The participants’ constipation scores were assessed before the intervention and on the sixth day using the CAS by a research assistant who was unaware of the study groups. In the group of abdominal massage with water, abdominal massage was carried out in eight steps using water. However, no complementary intervention was performed in the control group. All three groups received their medications prescribed by the nursing home physician.

In this study, the data were collected using a demographic information form and the CAS. The demographic form included information about age, gender, marital status, lifestyle (activity level, daily use of water, types of food, and daily use of tea), and history of diseases. This questionnaire was provided after review of the literature and consultation with experts. Its validity was confirmed after it was sent to the relevant experts and revised according to their comments. The CAS was designed by McMillan and Williams in 1989. ^34^ The content validity and reliability of the tool have been examined and confirmed in different studies. ^34 , 35^ In the study, for instance, the reliability (Intra-class correlation coefficients) of the tool was reported to be 0.97. ^35^ This scale consisted of eight items including ‘abdominal distension or bloating’, ‘changing in amount of gas passed rectally’, ‘less frequent bowel movement’, ‘oozing liquid stool’, ‘rectal fullness or pressure’, ‘rectal pain with bowel movement’, ‘small volume of stool’, and being ‘unable to pass stool’. This scale is a summated rating scale ranging from 0: no problems, 1: average problem, and 2: severe problem. The total score of the instrument could range from 0 to 16, where zero indicated the absence of constipation and 16 represented severe constipation. ^34^

The collected data were entered into the SPSS 22 software. After verification of normal distribution, the data were analyzed using descriptive and inferential statistical tests, such as chi-square, paired t-test, and ANOVA. P<0.05 was considered to be statistically significant.

The study was approved by the Ethics Committee of Shiraz University of Medical Sciences (IR.SUMS.REC.1397.698). The study was also conducted according to the Declaration of Helsinki. After the research objectives and methodology were explained, written consent forms were obtained from the patients. Moreover, they were ensured that they could withdraw from participation at any time if they were unwilling to cooperate.

## RESULTS

**Figure 1:**
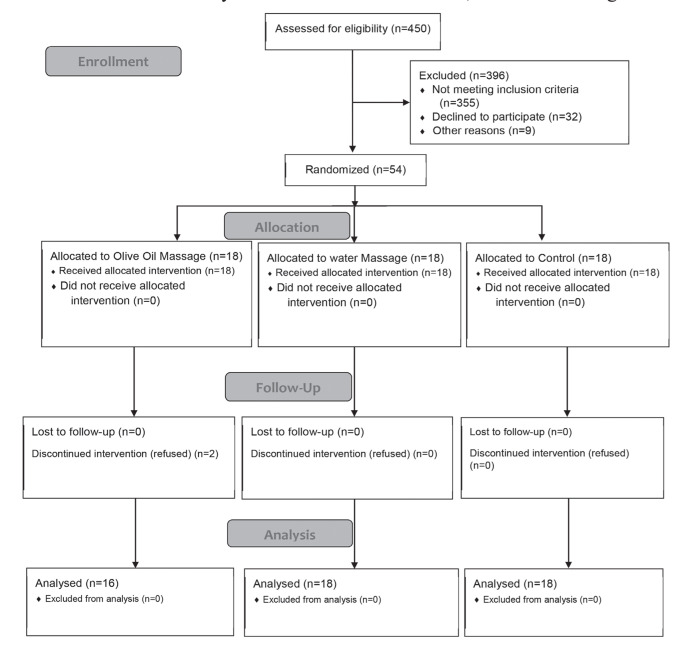
Figure 1: CONSORT flow chart of the participants

This study was conducted on 54 elderly individuals suffering from constipation who met the inclusion criteria. During the study, two participants in the group of massage with olive oil were excluded due to withdrawal from cooperation and discontinuing intervention (Figure 1). As two participants were excluded from the study, 52 individuals were entered into the final analysis. The participants’ age ranged from 60-93, with the mean age of 71.38±9.93 years. The results showed no significant difference among the three groups regarding the participants’ mean age (P=0.54). The results also revealed no significant differences among the three groups with regard to other demographic variables (Table 1). There was no statistically significant difference among the three groups as to lifestyle variables (activity level, daily use of water, types of food, daily use of tea and coffee, and daily use of fruits) (P>0.05).

**Table 1:** Distribution of the elderly people in the three groups in terms of demographic variables

		Massage with olive oil	Massage with water	Control	P value*
		N (%)	N (%)	N (%)	
Gender	Male	7 (43.8)	10 (55.6)	11 (61.1)	0.69
	Female	9 (56.3)	8 (44.4)	7 (38.9)	
Education level	Illiterate	2 (12.5)	8 (44.4)	4 (22.2)	0.74
	Primary school	7 (43.8)	3 (16.7)	4 (22.2)	
	Middle school	2 (12.5)	2 (11.1)	4 (22.2)	
	High school	3 (18.8)	3 (16.7)	3 (16.7)	
	Associate Degree	1 (6.3)	1 (5.6)	2 (11.2)	
	Bachelor’s or above	1 (6.3)	1 (5.6)	1 (5.6)	
History of smoking	Yes	3 (18.8)	5 (27.8)	4 (22.2)	0.91
	No	13 (81.3)	13 (72.2)	14 (77.8)	
History of medication use	Yes	5 (31.3)	9 (50)	7 (38.9)	0.52
	No	11 (68.8)	9 (50)	11 (61.1)	
Doing daily sports	Yes	4 (25)	0 (0)	2 (11.1)	0.06
	No	12 (75)	18 (100)	16 (88.9)	

* Chi-square test

The results demonstrated no statistically significant differences among the three groups regarding the mean score of constipation before the intervention (Table 2). After the intervention, however, a statistically significant difference was observed among the three groups in terms of the mean score of constipation. According to the LSD post hoc test, the constipation score of the massage with extra-virgin olive oil was statistically significant with the massage with water (P≤0.001) as well as the control group (P≤0.001). 

**Table 2:** Comparison of the elderly participants’ mean scores of constipation before and after the intervention in three groups

Group	Constipation		d score	Effect size	P value*
	Before the intervention Mean±SD	After the intervention Mean±SD			
Control	4.44±1.38	5.22±1.35	0.77±1.06	0.57	0.006
Massage with water	5.05±1.25	3.11±0.99	-1.94±0.93	1.72	0.02
Massage with olive oil	5.62±1.89	2.06±0.99	-3.56±1.26	2.35	≤0.001
P value**	0.87	≤0.001			

* Paired t-test;

** One-way ANOVA

As Table 2 shows, the mean score of constipation was 5.62±1.89 before the intervention, which decreased to 2.06±0.99 after that in the massage with extra-virgin olive oil group (P≤0.001). In the group of massage with water, the mean score of constipation was 5.05±1.25 before the intervention, which decreased to 3.11±0.99 after that, and the difference was statistically significant (P=0.02). The control group’s mean score of constipation was 4.44±1.38 before the intervention, which increased to 5.22±1.35 after five days, and the difference was statistically significant (P=0.006) (Table 2).

Analysis of the sub-items of CAS showed that there was a significant difference among the three groups of the study after the intervention regarding abdominal distension or bloating, change in the amount of gas passed rectally, rectal fullness or pressure, small volume of stool, and inability to pass stool (P<0.05) (Table 3).

**Table 3:** Comparison of the eight items of CAS before and after the intervention among three groups

Groups		Control	Massage with water	Massage with olive oil	P value*
Items		Mean±SD	Mean±SD	Mean±SD	
Abdominal distension or bloating	Before	0.62±0.61	0.79±0.63	1.17±0.62	0.04
	After	0.94±0.44	0.47±0.51	0.44±0.51	0.01
Changing in amount of gas passed rectally	Before	0.56±0.51	0.42±0.51	0.39±0.5	0.56
	After	0.5±0.51	0.16±0.37	0	0.001
Less frequent bowel movement	Before	0.75±0.68	0.89±0.58	1.05±0.54	0.007
	After	0.44±0.51	0.39±0.5	0.22±0.43	0.40
Oozing liquid stool	Before	0.62±0.25	0	0.11±0.32	0.34
	After	0.62±0.25	0	0	0.31
Rectal fullness or pressure	Before	0.75±0.44	1±0.57	0.72±0.57	0.25
	After	0.81±0.4	0.58±0.61	0.28±0.46	0.01
Rectal pain with bowel movement	Before	0.44±0.51	0.68±0.48	0.72±0.57	0.24
	After	0.69±0.6	0.47±0.51	0.28±0.46	0.10
Small volume of stool	Before	0.62±0.5	0.31±0.48	0.55±0.51	0.15
	After	0.87±0.72	0.74±0.45	0.33±0.48	0.02
Unable to pass stool	Before	0.62±0.5	0.68±0.48	0.83±0.51	0.48
	After	0.94±0.44	0.58±0.61	0.28±0.46	0.003

* Kruskal-Wallis Test

## DISCUSSION

Constipation is one of the most prevalent digestive problems among the elderly, which causes negative effects on their quality of life due to its chronic nature. ^12^ Evidence has demonstrated that the prevalence and severity of constipation were higher in old ages than in other periods of life. ^8 , 36^

The results of the present study demonstrated that the mean score of constipation decreased from before the intervention to after that in the abdominal massage with extra-virgin olive oil. The significant decrease in the constipation scores in this group in comparison with other groups indicated the effectiveness of the method in controlling constipation among the elderly participants.

Studies have demonstrated that abdominal massage was an effective method of treatment for constipation among the elderly. ^13 , 36^ For instance, a study performed on patients who were suffering from Parkinson’s disease revealed that abdominal massage for six weeks along with lifestyle training alleviated the symptoms of constipation. ^23^ In the same line, a study performed on patients who suffered from cancer showed that aromatic abdominal massage reduced constipation in these patients. ^26^ Similarly, another study reported that abdominal massage using rosemary oil, sweet lemon, black pepper, and sweet marjoram was effective in controlling constipation among the elderly individuals who were suffering from heart attack. ^37^ Abdominal massage with extra-virgin olive oil has many advantages over many medical methods, including low cost, absence of specific side effects, availability, and absence of interference with other methods. ^23 - 25^ When aromatic oil is absorbed by the skin, the body undergoes physiological, psychological, and pharmacological changes. Physiologically, this causes the individual to be relaxed and stimulated. Psychologically, it causes a reaction as the oil is smelt. Pharmacologically, it causes chemical and hormonal changes as the oil enters the blood. ^38^ Furthermore, the fact that the method is non-aggressive causes the individual to get along better with this type of treatment than with alternative methods, such as enema. ^39^ The use of complementary medicine methods in the treatment of patients with constipation has been recommended in previous studies. ^40 , 41^

In the simple abdominal massage group, the mean score of constipation decreased from before the intervention to after that. Although the decrease in the constipation score was significant, it was less than that in the massage with extra-virgin olive oil group. The study performed on patients hospitalized at special departments demonstrated that abdominal massage twice a day for three days reduced constipation in these patients. ^12^ The study performed on multiple sclerosis patients also revealed that abdominal massage accompanied with treatment advice had a positive effect on decreasing constipation in the patients. ^42^ In the same line, another study indicated that abdominal massage alleviated constipation in elderly people. ^13^ Other studies conducted in this regard have also shown that abdominal massage alleviated or reduced the symptoms of constipation. ^21 , 23 , 37^

In the control group, the mean score of constipation increased from before the intervention to after that. This implies that lack of complementary intervention caused the condition to deteriorate. As all three groups received their medical treatment, the differences between constipation scores can be attributed to abdominal massage as a complementary method.

Furthermore, as we found more decrease in the scores of patients in the abdominal massage with olive oil, it can be attributed to the absorption of oil via abdominal skin as well as facilitating massage than the simple abdominal massage group.

Using complementary medicine methods is one of the most efficient approaches for treatment of constipation in the present century. ^43^ Caregivers of the elderly people can help reduce constipation and increase their quality of life using complementary medicine methods. ^44^

One of the most important strengths of this study was conducting the intervention by using herbal oil used in Traditional Persian Medicine. The present study had some limitations which should be noted. This study was conducted on the elderly who were residing in the nursing homes. In addition, using only CAS for assessing constipation may not be comprehensive compared with combination with clinical examinations. Moreover, the biomedical mechanism of action for this procedure is not known. Finally, different laxative categories which were used by participants were not recorded.

## CONCLUSION

The present study demonstrated that abdominal massage using olive oil considerably alleviated the symptoms of constipation in the elderly individuals in comparison to the control group. Given the obtained results of the present study, healthcare professionals are recommended to be encouraged to apply this simple, inexpensive, and non-invasive method for controlling constipation among the elderly people who reside in nursing homes. Future studies are required to evaluate the eﬀects of abdominal massage with olive oil on constipation in other clinical conditions.
